# Valuing whole complex lives: Young adults’ experiences of recovery‐related principles in mental healthcare in the United States

**DOI:** 10.1111/hsc.12867

**Published:** 2019-10-03

**Authors:** Shannon Hughes, Robert Colbert, Ashley Baugh

**Affiliations:** ^1^ School of Social Work Colorado State University Fort Collins Colorado; ^2^ Department of Community and Behavioral Health Colorado School of Public Health Fort Collins Colorado; ^3^ An Enduring Love Co Fort Collins Colorado; ^4^ Children's Immunodeficiency Program Children's Hospital Aurora Colorado

**Keywords:** focus groups, holistic health, mental health, mental health recovery, patient preference, qualitative research, young adult

## Abstract

One in five young adults (aged 18–25 years) in the United States experiences a past year mental disorder, commonly including depression or anxiety. Yet, 1.5 million each year do not receive needed mental health services and are unlikely, in general, to seek formal mental healthcare. We aimed to inform the development of a novel programme for young adult mental health by first eliciting their positive and negative prior experiences with mental health providers. Four focus groups with 19 young adults (aged 19–26 years) recruited from the community and with moderate to severe depression and/or anxiety were conducted in 2018 in a western US state. Participants’ prior experiences with services/providers were elicited along six pre‐defined recovery‐related concepts: feeling listened to and validated, inclusivity, full information and consent, hope and optimism, connectedness, and change. Focus groups were audio‐recorded, transcribed verbatim and uploaded into NVivo version 12 software. Two independent coders used deductive thematic analysis to identify patterned responses. Feeling listened to and validated appeared as a cornerstone of other recovery concepts. Participants discussed past negative experiences with psychiatrists and regret for being put on medications in their teenage years without information or options. Hope and optimism were low because of a general focus by professionals to address immediate symptom‐based issues, rather than on improving their overall lives. Service providers’ focus on medication‐taking, and other one‐size‐fits‐all tools, was interpreted as lacking a sincere desire to help. Young adults were particularly sensitive to inauthentic interactions and superficial strategies, which left them craving care that incorporated their whole lives, acknowledged biopsychosocial interconnections and prioritised improving their lives over ‘feeling better’ in a given moment. Mental health providers should consider developing programmes that shift focus away from an exclusively medical understanding of distress and towards holistic, educational or relational approaches that value body, mind, self‐exploration and authentic connection.

**What is known about this topic?**

- Young adults often do not seek or access needed mental health services
- Relationship, respect and authenticity are important qualities of helping professionals according to young adults
- Unsupportive interactions with mental health professionals can fuel young adults’ isolation and hopelessness

**What this paper adds?**

- Young adults struggling with depression and anxiety report a preference for holistic care that acknowledges complex biopsychosocial interconnections
- Lack of information and individualised options, particularly with prescribed medications, contributes to hopelessness and disconnection
- Young adults value feeling listened to and validated, inclusion and informed choice, and opportunities for sustainable self‐learning and growth

## 1 INTRODUCTION

One in five young adults (aged 18–25 years) in the United States is reported to experience a mental disorder in the past year, with 9.3%–13.2% reporting a major depressive episode (Centre for Behavioral Health Statistics & Quality, 2015; Twenge, Cooper, Joiner, Duffy, & Binau, 2019). Relative to adult mental health, young people below age 21 years have been subject in recent years to significantly greater increases in the application of mental disorder diagnoses to their lives (Olfson, Blanco, Wang, Laje, & Correll, 2014). Rates of major depressive episodes and serious psychological disturbance increased by 63% and 71%, respectively, among young adults between 2009 and 2017 (Twenge et al., 2019). Yet, 1.5 million young adults who report needing mental health services do not receive them (Substance Abuse & Mental Health Service Administration, 2015). Young people are generally less likely to seek formal mental health treatment for myriad reasons, including cost, structural barriers, low perceived need and past unhelpful experiences with professionals (Wilson, Rickwood, & Deane, 2007). As rates of depression, anxiety and suicide continue to escalate among youth and young adults (Twenge et al., 2019), a pressing need exists to develop innovative approaches that can successfully engage this population and meet their specific needs and preferences. In this study, we aimed to inform the development of a novel programme for young adult mental health by first eliciting experiences related to their past positive and negative interactions with mental health providers.

Prior qualitative research with youth and young adults has confirmed the importance of well‐articulated recovery concepts, and identified additional needs specific to engaging this population. The recovery paradigm highlights how recovery from mental health challenges is an individualised journey of personal change, building a satisfying life and creating meaningful connections with the social world (Tew et al., 2012). Specific components of this journey include gaining power and control over one's life, cultivating social inclusion and a sense of belonging, meaningful personal relationships, hope and optimism about the future, and finding meaning and purpose in life. Recent systematic reviews of research incorporating young peoples’ voices emphasise the importance of relationship and engagement with helping professionals (Broad, Sandhu, Sunderji, & Charach, 2017), and building trust by adopting a non‐judgemental attitude, respect for privacy and an ‘authentic intention for caring’ (Kim & White, 2018). Other qualitative studies identify the desire to feel heard, validated and connected as priorities that shape young people's acceptance of and success in treatment (Issakainen, 2015; Wisdom, Clarke, & Green, 2006). In a grounded theory analysis of 524 blog posts from eight young adults, mental health struggles were described as an oppressive force that they should be able to control, but hopelessly cannot (Marcus, Westra, Eastwood, & Barnes, 2012). Prior unresponsive or unsupportive interactions with mental health professionals appeared to fuel these young bloggers’ feelings of profound loneliness and alienation.

The aim of the present study was to elicit the prior experiences of young adults struggling with mood‐related distress around how their interactions with mental health providers have successfully or unsuccessfully incorporated important recovery‐related concepts, such as feeling listened to and validated, meaningfully connected to others and instilled with hope and optimism. While prior research has illuminated the general needs and preferences of young adults, the present study more specifically examined the extent to which service providers have incorporated recovery‐related concepts from the perspectives of young adult service users. We further discuss implications of these results for programme development. Findings from this qualitative study were used to inform the planning of a novel mental health programme based on the lived experiences and voices of young adults. Evaluation findings from a pilot delivery of the programme are presently under analysis.

## 2 METHODS

### 2.1 Participants

Participants were 19 young adults between ages 19 and 26 years recruited from the community through social media advertisements, flyers and therapist referrals. Recruitment for these focus groups was part of a larger quasi‐experimental intervention study for young adults experiencing depression and/or anxiety. Participants who enrolled in the study, either in the intervention group or the comparison group, were eligible to volunteer for focus groups, which were conducted at baseline prior to the start of any intervention. Eligibility criteria for all participants included: aged 18–26 years, at least moderate depression and/or anxiety as measured by the Patient Health Questionnaire‐9 and Generalized Anxiety Disorder‐7, no active substance use in need of treatment as assessed by the short form of the Simple Screening Instrument for Substance Abuse (Centre for Substance Abuse Treatment, 1994), and no self‐reported suicide attempt in the prior 90 days.

### 2.2 Procedures

Four focus groups (*n* = 4, 5, 4, 6) were facilitated by either the first author or a qualified research associate. Focus group methodology was selected for the advantage of eliciting varied, and potentially sensitive, experiences that might only surface through the interaction of participants (Morgan, 2009). Focus groups took place between June and August 2018, prior to the start of the larger intervention study, and lasted approximately 75–90 min. Participants were provided a cover letter for consent and were told that they could leave the focus group at any time, without jeopardising their participation in the larger intervention study. Once all participants gave verbal consent, the audio recording device was turned on. Participants were instructed to avoid using their names, but were assured that transcriptions would be made anonymous regardless.

A focus group protocol guided the discussion while leaving room for participants to discuss other topics as they arose. The protocol started by asking participants to quietly reflect on the various mental health professionals and services they have engaged with in the past, and then describe to the group their earliest remembered experience receiving mental health help. The discussion then focused on describing prior experiences with getting help for a mental or emotional difficulty along six concepts. These concepts were written on a large poster board and read aloud to participants:

1. *Feeling listened to and validated*: feeling that others around you are really hearing what you have to say and validating your personal experiences in a non‐judgemental way;
2. *Inclusivity*: a sense that you can speak for yourself and that you are meaningfully included in decisions that affect your life;
3. *Full information and consent*: being provided with all relevant information about treatment options, meaningful opportunities to ask questions and discuss your options at any time, and the ability to consent or not consent to recommendations made by others;
4. *Hope and optimism*: an encouraging feeling that people can and do overcome challenges, barriers and obstacles that confront them;
5. *Connectedness*: a sense of belonging or purpose, and feeling connected to other people and the world and
6. *Change*: the experience of personal growth in yourself, or at minimum, a sense that positive change is possible and a belief that you can make it happen.

Participants were asked to describe prior experiences when one or more of these concepts was present or done well, and prior experiences when one or more of these concepts was absent or done poorly. With remaining time left, participants were asked to reflect generally on the best and worst qualities of the professionals and programmes they had engaged with in the past when seeking help.

Snacks and drinks were available to participants at the start of the focus group, and each participant received a $20 gift card to compensate for their time and travel expenses. This study was approved by the Colorado State University Research Integrity and Compliance Review office.

### 2.3 Data analysis

Audio recordings were professionally transcribed and uploaded into NVivo version 12 for coding and analysis (QSR International, 2018). Deductive thematic analysis was used to identify patterned responses within the six broader recovery‐related concepts that guided the focus group discussion (Braun & Clarke, 2006). Two independent coders first read all four focus group transcripts and then coded one transcript by applying the a priori concepts to segments of text. Individual extracts of text may have been coded once or coded multiple times insofar as they were relevant to one or more of the six recovery‐related concepts. The two coders and the first author met to examine inter‐coder agreement from this initial coding stage and discuss emerging patterns in semantic content found within each of the broader conceptual groupings. All three coders agreed on the development of initial themes, capturing the range of positive and negative experiences described by participants, within each conceptual grouping. The two coders then independently coded the remaining three transcripts, maintaining the flexibility to add emergent themes as they progressed in the coding progress. Finally, the two coders and the first author met again to examine inter‐coder agreement, discuss any newly emerging themes, and review and refine the final set of themes with regard to arriving at the most accurate representation of the range of experiences within each recovery‐related concept.

## 3 FINDINGS

### 3.1 Sample description

Table 1 provides the demographic characteristics for this sample of young adults. Most participants identified as female (78.9%), White (84.2%) and with at least some college education completed (94.7%). Most were employed full time (63.2%) and less than half were current college students (47.4%). In their baseline assessments, participants reported first receiving a mental health diagnosis at a mean age of 16 years (min 12; max 21), most often related to depression or anxiety. In all, 14 participants (73.7%) reported a history of psychotropic medication use, having tried an average 2.5 (*SD* = 2.03) prescribed medications. The mean age of starting on a psychotropic medication was 17 years (min 11; max 21), with a combined 41 person‐years spent on psychiatric medications.

**Table 1:** Demographic and clinical characteristics of young adults, *n* = 19

	*N* (%)
Sex, female	15 (78.9)
Race/Ethnicity	
Black or Hispanic	2 (10.5)
White, non‐Hispanic	16 (84.2)
Not reported	1 (5.2)
Education	
High school	1 (5.2)
Some college	16 (84.2)
Bachelor's degree	1 (5.2)
Graduate degree	1 (5.2)
Marital status	
Married	2 (10.5)
In relationship	8 (42.1)
Single	9 (47.4)
Student status	
Student	9 (47.4)
Non‐student	10 (52.6)
Employment status	
Full time	12 (63.2)
Part time	4 (21.1)
Unemployed	3 (15.8)
Prior use of at least one psychotropic medication	14 (73.7)
Prior diagnoses^a^	
Anxiety disorder	10 (52.6)
Depressive disorder	7 (36.8)
ADHD	5 (26.3)
PTSD	3 (15.8)
Bipolar 1	1 (5.2)
Anorexia/Bulimia	1 (5.2)
Borderline PD	1 (5.2)
No prior diagnoses	6 (31.6)

a Percentages sum to >100% due to multiple diagnoses per person.

Most participants (78.9%) described accessing therapy or counselling services at some point in their lives. Of those, five reported that their first experience was involuntary or mandatory. Participants also reported seeking help from a psychiatrist or doctor, non‐emergency hospital visits, college campus resources, or talking with family, teachers or youth pastors. A few participants (21.1%) described accessing what they considered to be alternative or complementary care services, including brain spotting (i.e. a therapy to access unprocessed trauma through points in a person's visual field), EMDR or acupuncture.

### 3.2 Listened to and validated

Feeling listened to and validated was the most discussed concept of the six and appeared to be a cornerstone of other concepts, particularly inclusivity, full information and consent, and connectedness. Participants described positive experiences as being able to talk openly to professionals without fear of judgement or paternalistic responses. Participant B4 explained, ‘I think always the best thing is just listening and being able to trust the person…without worrying that they're going to judge you or kind of make a decision for you’. This concept also extended to a felt sense of genuineness from a professional or that they truly ‘want to be there for you’ (B4).

Participants also described negative experiences with professionals and family members who ‘brushed off’ the seriousness of their distress with rote responses that lacked true understanding. As young people, and especially as struggling adolescents, participants described being unsure about what they were truly feeling or how to articulate it to others. Being brushed off by family members or professionals as ‘a moody teenager’ or ‘going through a phase’ exacerbated their sense of isolation and having to ‘go it alone’. Other participants explained that no one ever inquired about what was going on in their lives or demonstrated a desire to genuinely listen to them. These experiences further impacted their sense of connectedness, as described by a participant who was referred to her school counsellor:

She never really talked to me about what was going on in my life, why I was feeling this way, it was more like, “Hey you need to come here and do your homework, and if you don’t do your homework you’re not going to get into college” kind of stuff… it was like that wasn’t the deeper issue. They didn’t connect on what was actually going wrong. (E4)

### 3.3 Inclusivity

Participants discussed the concept of inclusivity in two main ways. Primarily, inclusivity meant feeling as if they were part of the conversation and included in decisions that would affect them. Participants reflected on their experiences with inclusivity in mostly negative terms when, as adolescents, the adults and professionals in their lives would talk over them or around them, thereby fostering a sense of not being in control of their lives or care. For example,

When I was younger my mom would always be there whenever my family doctor would ask me about my therapy or anything and he would listen to her more than he would listen to me. Which it's like not about her and she's not the one going to therapy so it made it hard when he's prescribing medicine or stuff like that cause he wasn't really listening to what I was saying. (D4)

Another participant described adults in her life over‐reacting to a situation and immediately taking away her sense of control and inclusion in important decisions:

I was writing and my dad found something in my journal that he took kind of as like something he needed to be concerned about. So he took it to my therapist and she ended up making me sign something for basically being put on suicide watch. And it was something that she didn't listen to me explain it, and I wasn't really having any big issues at that time. And I just didn't really feel like I was included in the decision. (B4)

The second way young adults elaborated on inclusivity was in terms of having individualised options presented to them. This was contrasted with experiences of professionals offering whatever single tool they seemed to most prefer, which was interpreted as them having ‘an agenda’. Participants specifically described feeling annoyed by continued suggestions from therapists to practice ‘mindfulness’, despite repeated explanations that mindfulness was not working or was not what they needed. Participant A1 explained their experience with this as, ‘oh, mindfulness. They talk about mindfulness so much and I swear I was like, I hate mindfulness. Like, that's not what I need and I just—I don't know like that's been a very big struggle for me…’ Instead, participants valued having individualised options and the quality of adaptability on the part of the professional ‘to think on your feet and be creative or be out of the box’ (B3).

### 3.4 Full information and consent

Participants discussed the concept of full information and consent mostly in the context of their experiences with psychiatrists, which were overwhelmingly negative in tone. Receiving no information on treatment options appeared as a significant point of strife and regret for some participants:

I feel like I wasn't actually given very much information about therapy options and stuff at that point. They did just go with like the medication. I feel like there were probably better options… Rather than just kind of like, I don't know shutting a certain part of my brain off artificially so I would just kind of go through the day and be okay with everything. (A4)

When we went into the doctor, the doctor listened to me, it felt like, however, she didn't really offer another option,… She went kind of straight to medication. Which, I feel like, I would definitely take that back. (A2)

Other participants described feeling troubled by not receiving any information about the drug prescribed to them or feeling like a ‘lab rat’ through the trial‐and‐error process of finding a drug that worked well for them:

And depending on what psychiatrist you meet with, the information they give you is different like everybody believes a different thing like what medication you should be on and how the medication works… I feel like I’m just getting the wrong information so I think I need to go and look it up myself. I don’t know if they’re not educated or they don’t think I would understand what they’re saying… (A1)

When I went to the doctor and they were like, “Oh, let’s try this medication” and they didn’t tell me all of the options, they just picked one and were like, “Here, take this and see what happens” and then that one didn’t work and so then they tried another one and were like, “Here, try this one,” and they didn’t tell me all the possible options at the beginning and help me to choose it. (D1)

On the positive side, young adults reported having trust in professionals who can say ‘I don't know, but I can look into some things and get back to you’ (B3), or who lay out all the options so that they can come to their own informed decision. Participant E4 shared a helpful interaction with their therapist where ‘she just drew it out of me and then gave me advice and laid it all out for me, like a path, paths that I could take. And she didn't say her own opinion, she just laid it out. That just really helped me to see it clearly’.

### 3.5 Hope and optimism

Feelings of hope and optimism were discussed in the context of grappling with the question, ‘Why do I feel the way I do?’ Some participants shared their sense of confusion around making sense of their distress when their rational minds would tell them that they have nothing to complain about: ‘You start thinking you're crazy for feeling these things. Especially like, I don't know, being a white female… I definitely am constantly like, Why do I feel like I have issues?… I have nothing to complain about’ (A2). Participants acknowledged the impacts of oppression and trauma as legitimate experiences to feel depression and anxiety over. In the absence of direct oppression and trauma, however, there was no apparent reason to live with such negative feelings. This further led to a sense of isolation and hopelessness, as if they had to ‘deal with it myself’ and ‘figure it out’ on their own.

Other young adult participants noted how their hope and optimism are low because of a general focus by professionals to address immediate symptom‐based issues, rather than on improving their overall lives. Participant A2 explains this sentiment:

… honestly I have a hard time really with anybody about the hope and optimism just because … it's never been, "You can get better!" It's more like "How do we overcome this that you're dealing with right now to overcome the next thing?”

### 3.6 Connectedness

Connectedness emerged as an important concept that closely hinged on feeling listened to and validated, and extended into a desire for belonging, depth in their interactions with others and understanding mind–body connections. Participants described their most successful experiences with counselling centres and therapeutic groups in terms of the sense of community that was cultivated. Participants described feeling ‘connected to people on a level that you can't just get to every day when they're complete strangers’ (C4).

In their interactions with therapists and doctors, participants experienced the greatest connection when they felt as if they were ‘more than just a client’, which could be demonstrated with small acts, such as the professional offering relevant books or resources based on conversation shared in a prior session. Participants equally discussed negative experiences with therapists or professionals who failed to demonstrate their dedication, as reflected by participant B1, ‘I just felt sometimes, they're there for you in the moment but then they're not… You're just a patient to them and they don't understand. They don't care’.

Young adults in this sample were particularly sensitive to inauthenticity and superficiality on the part of professionals. They expressed a desire for depth in their interactions and in the solutions offered to them. Even while having a positive relationship with her therapist, participant C4 reflected:

I just didn't connect as well with her and I felt like some of the coping mechanisms and stuff that she gave me were good, but they were very surface level and they didn't get down to the root of my problems. Especially now that I'm farther along in that path, I realize that it was really surface level.

Prior superficial interactions seemed to leave young adults craving care that integrated into their whole lives and acknowledged mind–body connections: ‘I feel like when people look at you holistically, it works better than just treating your brain, your mental health or just like your body, because it is all one’ (A1). Some participants expressed exasperation at professionals’ focus on treating symptoms of their depression to the neglect of other possibilities, for example,

… something else can be wrong with your body that can cause depression like me, having cyst on my ovaries… It can be an underlying hormone problem, it could be a lack of a vitamin, it could be anything. It’s not all just mental illness and – because a lot of people jump to depression and be like, ‘Oh, you’re depressed.’ I’m like, ‘It could be something totally different (C1).

Connectedness thus emerged as spanning a sense of community and belonging, and further, to an expressed desire for depth, integration and holism from treating professionals.

### 3.7 Change

Participants discussed change as an elusive concept that can be difficult to notice or track. They noted that tracking change could be a helpful role for therapists and professionals. One participant (C2) explained, ‘… because it's gradual… it is hard to track that (change) yourself. So it's good to see, to have other people see that change and to comment on it’. Participants who had less prior experience with therapy or treatment described having to ‘figure out things on my own’ (A2), which led to questioning their self‐efficacy, for example, ‘I don't know if I can change… the anxiety and thoughts that I have on a daily basis, from the moment I wake up until I go to bed’ (B2).

Participants described wanting ‘real’ change versus superficial change and were frustrated by experiences when they felt compelled to ‘fake the change’ to gain a sense of autonomy or avoid letting others down. For example,

Every single person that I've seen, they start out in the beginning saying like, "What do you hope to change?" And then, at the end, they talk to me like, "Well what do you think has changed and do you feel better?" And there's this pressure that I have to say, "Yeah I'm better." And just because I don't want to be there anymore… I fake the change. (A2)

‘Fake’ change was also used in the context of superficial or inauthentic change, contrasted with: ‘Something that is sustainable that I can do for the rest of my life instead of just like something I can only do for a short period of time… something that is just my way of being. That like eventually just becomes how I am’ (B3). Real change is not a quick fix, but rather a way of living life as a responsible, choice‐making person:

I feel like I'm responsible… I'm in control. Versus like, yeah, having a label. A mental health label or a disability label or a pill that I'm supposed to take that, you know… Then it makes the prescription responsible. I take the pill so that's supposed to make me feel better… I want to be the one in control of making myself feel better (B3).

## 4 DISCUSSION

Young adults in this study described themselves as whole persons with complex lives. They expressed desire for mental healthcare that acknowledged interconnections and prioritised improving their lives over ‘feeling better’ in a given moment. Feeling listened to and validated appeared as a cornerstone of other recovery concepts. Participants discussed full information and consent primarily in the context of past negative experiences with psychiatrists. Receiving no information on treatment options appeared as a significant point of strife and regret for participants who were prescribed psychotropic medications in their teenage years. Young adults noted that a general focus by professionals to address immediate symptom‐based issues, rather than on improving their overall lives, contributed to their low hope and optimism for the future. One‐size‐fits‐all tools, such as medications or mindfulness that were sometimes ‘pushed’ by professionals, frustrated participants and were interpreted as the professional lacking a sincere desire to help. Young adults were particularly sensitive to inauthenticity and superficial interactions. This left them craving care that incorporated their whole lives, acknowledged complex mind–body connections and offered personalised guidance to support sustainable learning and growth.

These findings are consistent with what prior research has found on young adults’ preferences and experiences engaging with healthcare services. Important qualities of a helpful relationship with an adult include respect, time shared, openness, authentic relationship‐building and guidance (Martin, Romas, Medford, Leffert, & Hatcher, 2006). Young people, in general, report preferences for non‐medication strategies for supporting their mental health (Hickie, Luscombe, Davenport, Burns, & Highet, 2007; McCann & Lubman, 2012; Raue, Schulberg, Heo, Klimstra, & Bruce, 2009). In busy and overtaxed practice settings, the application of these ideals is complicated by organisational realities of time constraints, high workloads and staff burnout (Kim & White, 2018). Meaningful improvements in young adult mental health might therefore require systemic mental healthcare reform so that young adults’ feelings of alienation and hopelessness are not exacerbated by the act of help‐seeking (Marcus et al., 2012).

### 4.1 Limitations

Young adults in these focus groups were homogeneous in their demographic characteristics and are unlikely to represent the experiences and preferences of demographically and socioeconomically diverse young adults. Persons experiencing more extreme states, such as psychosis, are also not represented in this study sample and findings should not be generalised to young adults with more serious mental and emotional distress. Participants in this study were also seeking to enrol in a novel intervention programme and, therefore, might have greater negative prior experiences with conventional mental healthcare than is typical. However, a range of experiences and symptom severity appeared to be represented across participants. For example, a few participants had no prior mental health diagnosis and reported few lifetime interactions with formal mental health services outside of a University counselling centre. Other participants reported multiple diagnoses, inpatient hospitalisations and long histories of medication‐taking. Regardless, the experiences of this largely white, female, fairly educated sample of young adults might not generalise to the potentially diverse experiences of persons from marginalised communities. At the same time, young adult participants were eager to share their voices and provided rich data for programme planning purposes.

### 4.2 Application to programme planning and development

The experiences and voices of young adults should push mental health providers to consider developing programmes that shift focus away from an exclusively medical understanding of distress and towards holistic, educational or relational approaches that value body, mind and authentic social connection. Programmes should emphasise the whole biopsychosocial person and avoid symptom‐based solutions, offer multiple options and sustainable strategies for personal growth, and provide non‐judgemental space for the process of making meaning out of mental and emotional distress.

More holistic and integrated models can be found in Coordinated Specialty Care and Open Dialogue approaches for first‐episode psychosis. Both models use team‐based and person‐centred approaches to support multiple aspects of a person's life, with Open Dialogue adopting an explicit aim to minimise the use of psychotropic drugs (Seikkula et al., 2006). However, such coordinated and comprehensive support is not often available to young adults with milder forms of psychological distress. Clinicians in routine practice might therefore adopt stepped care approaches and encourage the generation of greater psychosocial options (Murphy, McCarthy, Baer, Zima, & Jellinek, 2014). For example, education around sustainable adjustments to diet, nutrition and exercise could be offered as biological points of intervention for depression and anxiety. Ample research evidence documents the value of diet patterns and nutritional support in mental health (Akkasheh et al., 2016; Jacka et al., 2017; Rucklidge, Kaplan, & Mulder, 2015), and recent research in humans suggests a role for gut microbiota in depression (Valles‐Colomer et al., 2019). Exercise for depression has demonstrated efficacy equivalent or superior to pharmacological and psychological interventions in multiple randomised controlled trials (Cooney et al., 2013).

Physical interventions described above coupled with other first‐line psychosocial approaches, such as peer support and ‘social prescribing’, might together offer valuable treatment packages for young adults experiencing depression and anxiety. Peer support modalities prioritise principles articulated by young adults to be important, including meaningful connection, authenticity and sustainable growth and healing. Peer‐based interventions, such as Hearing Voices Groups and Alternatives to Suicide Groups, are spreading in the United Kingdom, the United States and other countries as an alternative discourse to narrowly focused psychiatric narratives of distress (Dillon & Hornstein, 2013). *Intentional Peer Support* provides trainings internationally to service users and professionals on how to build partnership, create dialogue and practice ‘the art of connection’. Benefits of peer support modalities have been described as fostering a shared sense of identity, increased self‐confidence, social inclusion, resilience and self‐agency (Faulkner & Basset, 2010; Seebohm et al., 2013).

In a similar vein to ‘social prescribing’ in the United Kingdom (Chatterjee, Camic, Lockyer, & Thomson, 2017), mental health programmes for young adults might consider supporting self‐exploration and social connections as additional first‐line approaches. A variety of positive outcomes related to self‐esteem, psychosocial competence and maturity, connectedness, positive identity, and life satisfaction appear to be mediated by a process of identity exploration and formation that can be fostered by engagement in self‐defining activities (Coatsworth, Palen, Sharp, & Ferrer‐Wreder, 2006; Tew et al., 2012). Programmatically, flexible‐spending accounts can be applied for activities of young adults’ choosing, such as gym or sports membership, meditation, martial arts, music or art lessons, or outdoor excursions.

Young adults see themselves as interconnected, whole persons and report a desire for treatment options that explore and engage their full and complex lives. When it comes to young adult mental health, at minimum, professionals should listen, validate and facilitate informed choice. In the process of programme development, planners should *start with* the building blocks of validation, authentic connection and choice, and ask what kind of environment or programming would be most conducive to fostering these values and ways of being. This requires thinking beyond the medical model of distress towards holistic, sustainable self‐development approaches.

## CONFLICT OF INTEREST

The authors report no conflicts of interest.
